# Complexity, rate, and scale in sliding friction dynamics between a finger and textured surface

**DOI:** 10.1038/s41598-018-31818-3

**Published:** 2018-09-12

**Authors:** Behnam Khojasteh, Marco Janko, Yon Visell

**Affiliations:** 1 Department of Electrical and Computer Engineering, Media Arts & Technology Program, and Department of Mechanical Engineering, University of California, Santa Barbara, California, 93106 USA; 2 Department of Electrical and Computer Engineering, Drexel University, Philadelphia, 19104 USA

## Abstract

Sliding friction between the skin and a touched surface is highly complex, but lies at the heart of our ability to discriminate surface texture through touch. Prior research has elucidated neural mechanisms of tactile texture perception, but our understanding of the nonlinear dynamics of frictional sliding between the finger and textured surfaces, with which the neural signals that encode texture originate, is incomplete. To address this, we compared measurements from human fingertips sliding against textured counter surfaces with predictions of numerical simulations of a model finger that resembled a real finger, with similar geometry, tissue heterogeneity, hyperelasticity, and interfacial adhesion. Modeled and measured forces exhibited similar complex, nonlinear sliding friction dynamics, force fluctuations, and prominent regularities related to the surface geometry. We comparatively analysed measured and simulated forces patterns in matched conditions using linear and nonlinear methods, including recurrence analysis. The model had greatest predictive power for faster sliding and for surface textures with length scales greater than about one millimeter. This could be attributed to the the tendency of sliding at slower speeds, or on finer surfaces, to complexly engage fine features of skin or surface, such as fingerprints or surface asperities. The results elucidate the dynamical forces felt during tactile exploration and highlight the challenges involved in the biological perception of surface texture via touch.

## Introduction

When we explore objects via touch, complex frictional forces are produced by means of which we are able to perceive surface features, to identify the object at hand, and to facilitate grasping or manipulation. In contrast to vision and audition, the origin of tactile signals, which arise mainly through contact between the skin and touched objects, is unclear. These interactions produce dynamic mechanical deformations that span multiple length and time scales shaped by geometry and mechanics of the touched surface and heterogeneous hand tissues. The resulting stress patterns excite activity in thousands of mechanoreceptive nerve fibers that innervate the skin, translating mechanical stresses into temporally and spatially precise neural information^1^. The variation in these patterns with movement make it possible to infer properties of touched surfaces, including roughness^2^, shape^3^, adhesion^4^, texture^1^, bumps and edges^5^.

Because of the complexity of finger-surface interactions, it is difficult to relate mechanical signals felt by the finger to the surface being sensed. Sliding contact between the finger and a textured surface, even one that is macroscopically smooth^6^, yields rapid stress fluctuations^7,8^ that can propagate widely in the skin^9^. The established duplex theory of texture perception indicates that temporal variation in friction forces, rather than spatial variations, dominate the perception of fine surface textures^8^. However, the manner in which time-varying forces produced during tactile sliding encode surface properties is not fully understood. Ameliorating this could elucidate the mechanisms of touch perception, and inform the engineering of new technologies for tactile sensing and feedback.

The difficulties in understanding these mechanical interactions can be traced to the nonlinear constitutive behavior of fingertip tissues^10,11^, the complexities of frictional phenomena^12^, including the moisture content of the fingertip skin^12–14^, stick-slip transitions^15–17^, the presence of non-Coulombic regimes^18^, effects of fingerprints^19,20^. and surface shape induced skin deformations. Thus, during sliding contact of the skin with even simple surfaces such as sinusoidal gratings^6,21,22^, or braille dots^23^, forces fluctuate greatly in time. Existing mechanical models based on simplified^24^ or continuum biomechanics^25,26^, or signal models, including nonlinear autoregressive processes^6^, are unable to fully account for the forces produced during tactile sliding, or relate them to surface properties or other interaction parameters. Numerical simulations often involve unreasonable simplifications, omitting physical effects like the aforementioned^27,28^, and are difficult to compare with measurements, limiting their utility for explaining tactile interactions.

In this study, we sought to ascertain whether the scale- and rate-dependence of friction force fluctuations during tactile sliding could be explained via a numerical model that accounts for the main geometric and mechanical features of the finger, the counter surface, and their contact. We investigated contact forces that were produced during sliding of real fingers on textured contact surfaces with known geometry, and simulated forces based on finite element method (FEM) simulations with equivalent surface shapes and sliding speeds. These simulations accounted for tissue geometry and heterogeneity, dissipation, deformation, hyperelasticity, and interfacial adhesion. We compared the numerical results with experiments for different surface wavelengths, scanning speeds, and contact conditions. Because of the complex dynamics involved in these interactions, we analyzed measured and simulated frictional forces in the time, space, and frequency domains using linear and nonlinear methods, including recurrence plot analysis^29,30^. The results revealed that the model has greater predictive power for higher sliding speeds and for the coarser surfaces. Based on these discrepancies, and the analytical results, we identified key factors affecting frictional forces produced at different scales and regimes. We discuss implications of the results for understanding human abilities of surface texture perception.

## Results

**Figure 1:**
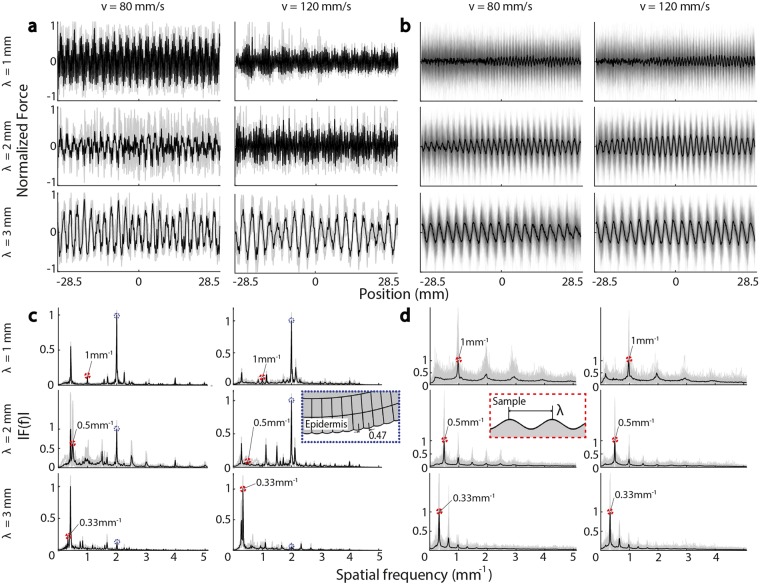
Spatial patterns of (**a**) simulated and (**b**) measured frictional forces (gray lines: individual trials, black lines: ensemble average) at two velocities (80, 120 mm/s) and three surface wavelengths (1, 2, 3 mm). While the force patterns qualitatively reflect the surface period, there was greater variance in the measured force data. The spatial spectra of (**c**) simulated and (**d**) measured forces exhibit prominent peaks, at both harmonic and mixing frequencies. Prominent frequency components associated with the surface period and the epidermal ridge spacing are both present.

We compared a total of 528 time-resolved friction force trajectories *F*_*T*_(*t*) produced by 9 different index fingers sliding on sinusoidally textured surfaces with 60 simulated force trajectories. The measured and simulated force trajectories were produced in six conditions that varied in sliding speed (*v* = 80, 120 mm/s), and the wavelength (*λ* = 1, 2, 3 mm) of the sinusoidal surface height function *h*(*x*). For analysis, we converted forces to the spatial domain, *F*_*T*_(*x*), using the tracked location *x*(*t*) of the finger (see Methods). Repeated trials in simulated conditions began from different initial conditions. The results revealed similarities between the measured and simulated force trajectories at both velocity levels and all three texture wavelengths. In all cases, forces exhibited fluctuations due to interactions between the epidermal ridges (i.e. fingerprints) and the sinusoidal surface, and concomitant rapid changes in contact (Fig. 1). The ratio of the standard deviation to the signal amplitude was largest for the measured force data, indicating that there was greater variance in the experiments than in the simulations.

### Spatial Frequency Content

The magnitude spectra *A*(*f*) of force fluctuations exhibited power law variations with surface frequency *f*, that is *A*(*f*) ~ 1/*f*^*α*^, as expected in sliding friction interactions and observed in prior studies^31^. The spatial patterns of frictional forces reflected the periodicities of the textured samples, exhibiting faster oscillations at smaller surface wavelengths, *λ*. The oscillations were quasiperiodic, varying in phase and amplitude within and between trials (Fig. 1a,b).

The trial to trial variance in the spatial spectrum of forces was smaller for the simulated data than the measured data (Fig. 1c,d), indicating that variations in simulation initial conditions, coupled with tissue and contact mechanics, were not sufficient to reproduce force variations as large as those that we empirically measured. This can be partly attributable to the multiple real fingers represented in the data set, but may also be because the model is effectively smooth at the microscale, in contrast to real surfaces.

The spatial spectra of both simulated and measured signals exhibit prominent maxima at spatial frequencies corresponding to integer multiples of the fundamental spatial frequencies (*λ*^−1^ = 1, 0.5, 0.33 mm^−1^). The simulated forces produced by the finer surfaces (*λ* = 1, 2 mm) exhibit an additional maximum with a frequency of approximately 2 mm^−1^, which we could associate with the 0.47 mm spacing of the epidermal ridges. No similar frequency manifested in the measured force patterns. As discussed below, this difference is likely attributable to the parallel configuration of fingerprint ridges in the model, which contrast with the varying ridge orientation of real fingers. As analyzed in the supplementary information (Supplementary Data S5), and further discussed below, we associated this, and frequency splitting in the spectrum for the *λ* = 3 mm, *v* = 80 mm/s condition, with partial out-of-phase ridge motion, which could occur due to the effectively parallel ridge configuration in the model. This did not appear in the measurements, where ridge orientation varies across the fingertip.

### Recurrence Patterns

**Figure 2:**
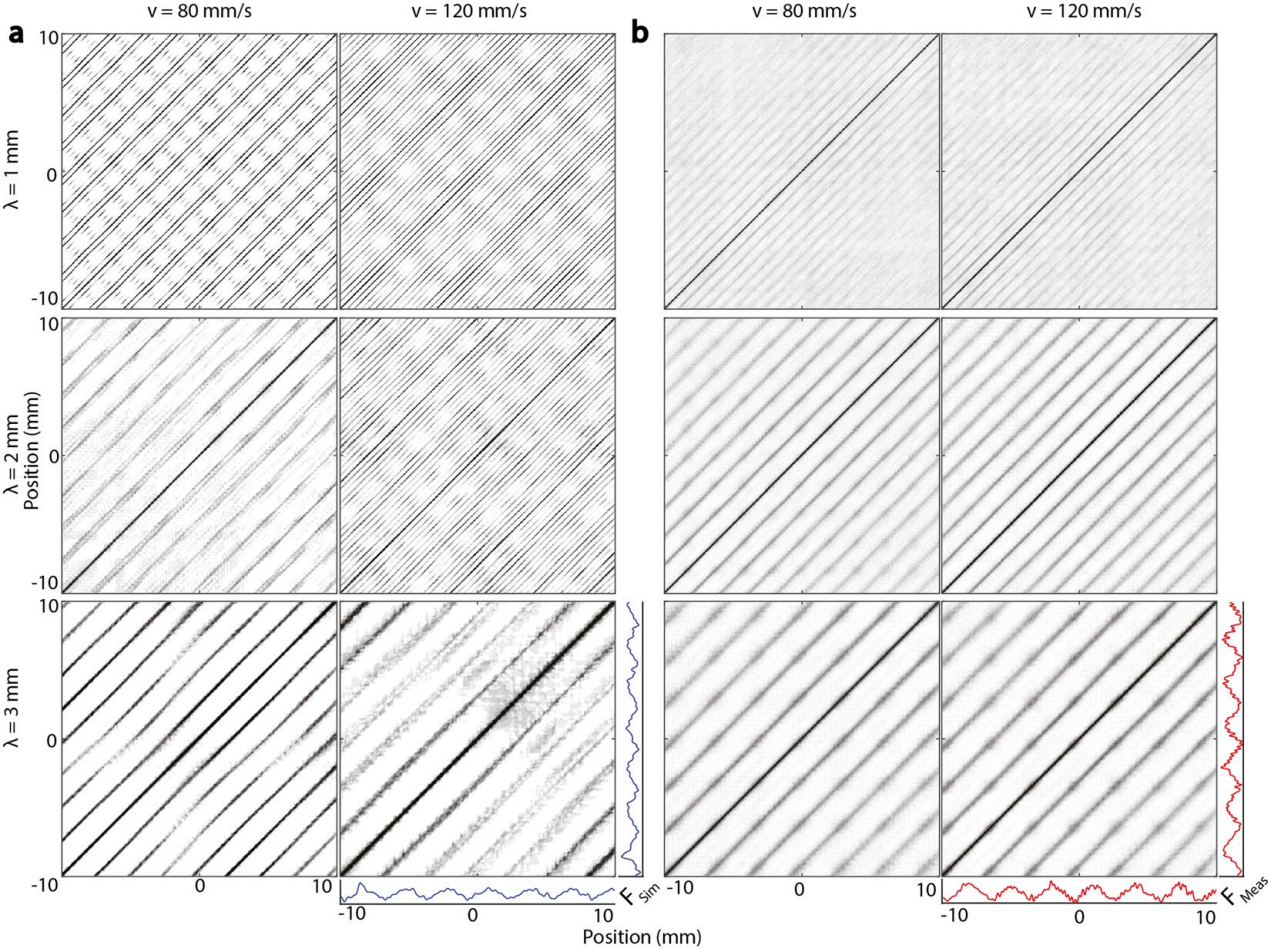
Mean recurrence plots of (**a**) simulated and (**b**) measured forces in all conditions. The diagonal lines reflect the periodicity of the force signals, illustrating the large qualitative agreement between simulated and measured force patterns. Measured and simulated forces exhibited greater regularity for coarser surfaces. A quantitative comparison is made through the joint recurrence analysis presented below.

We used recurrence analysis to capture the nonlinear correlations in the signals (see Methods), yielding recurrence matrices ${{\bf{R}}}_{ij}(\varepsilon )={\rm{\Theta }}(\varepsilon -\Vert {{\bf{x}}}_{i}-{{\bf{x}}}_{j}\Vert )$, and their ensemble averages ${ {\mathcal R} }_{ij}=\overline{{{\bf{R}}}_{ij}}$. The latter captured similarities in the force trajectories at different relative phases across many trials, revealing conserved patterns of quasiperiodic behavior. The prominent diagonal lines reflect self-similarities in the dynamical state at different times across trials for simulated or measured data (Fig. 2), and their spacing *δ* along the opposing diagonal reflect intervals over which the dynamical state approximally recurred. For both measured and simulated data, in three of the conditions (*λ* = 2 mm, *v* = 80 mm/s; *λ* = 3 mm, *v* = 80, 120 mm/s), the line spacing was close to the wavelength of the sample (*δ* = 2, 3, 3 mm respectively). The value of *δ* was shifted lower for the *λ* = 3 mm, *v* = 80 mm/s condition in agreement with our finding (discussed above) that the corresponding spectral line was shifted to higher frequencies (Supplementary Data S5).

In the remaining three conditions, which included slow or fast sliding on fine (1 mm) surfaces and fast sliding on medium (2 mm) surfaces, the simulated force trajectories included a contribution from lines with fine spacing, *δ* ≈ 0.47 mm, matching that of the epidermal ridges, in agreement with the spectrum analysis (as noted in the foregoing).

**Figure 3:**
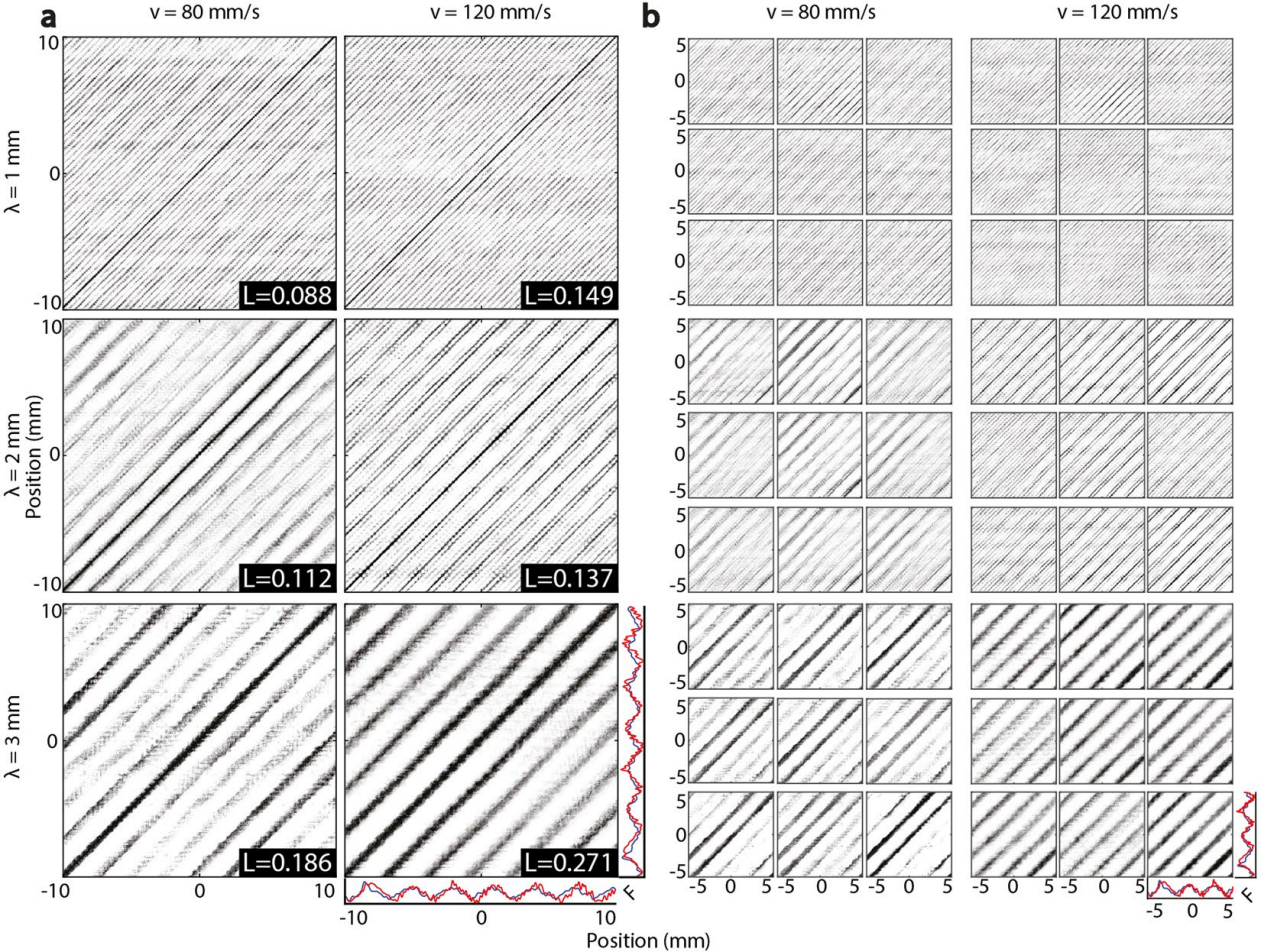
Mean JRPs of (**a**) all 880 combinations as well as (**b**) subject-specific measured and simulated trial pairs. The spacing of diagonal structures reflects the fundamental period of forces, and resembles that of the surfaces. While minor differences in the force patterns of the participants exist, the friction dynamics characteristics in each condition are preserved. The quantification analysis yielded mean recurrence lengths *L* indicating greater agreement between simulations and measurements at higher speeds and longer wavelengths.

We further quantified the similarity of measured and modeled forces via ensemble averages ${{\mathscr{J}}}_{ij}$ of joint recurrence plots ${{\bf{J}}}_{ij}={\rm{\Theta }}({\varepsilon }_{{\bf{x}}}-||{{\bf{x}}}_{i}-{{\bf{x}}}_{j}||)\cdot {\rm{\Theta }}({\varepsilon }_{{\bf{y}}}-||{{\bf{y}}}_{i}-{{\bf{y}}}_{j}||)$ over all 880 combinations of computed vs. measured forces in matched conditions *λ*, *v*. The prominent diagonal line features revealed high degrees of similarity between measured and simulated force trajectories even over long distances (Fig. 3), indicating that the geometry and mechanics that are represented in the model reproduced force trajectories similar to those that were empirically observed. The line distance *δ* increased with *λ*, and the line orientation was almost exactly *θ* = *π*/4 from vertical in all conditions, indicating that the simulated and measured forces were similarly quasiperiodic.

We analyzed the JRP data using standard recurrence quantification analyses, based on line length statistics (Table 1), which measured the mutual similarity of the measured and simulated forces at relative times. Mean line length *L* was larger at high sliding speed (*v* = 120 vs. 80 mm/s) for every wavelength (*λ* = 1, 2, 3 mm). Surprisingly, this indicates that the model had greater predictive power at higher speeds, when forces fluctuated more rapidly in time, than at lower speeds (mean $\overline{L}$ = 0.13 vs. 0.19 for *v* = 80, 120 mm/s), albeit with somewhat greater variability (resp. mean $\overline{{\sigma }_{L}}$ = 0.055 vs 0.085). The results also show that, at both speeds, simulated and measured forces became most similar for the longest surface periods.

**Table 1:** We employed a standard method of quantifying signal similarity via joint recurrence plots (JRP), based on the statistics of line lengths *L*, yielded higher mean values at higher speeds *v* and larger wavelengths *λ*, and reflecting the typically greater similarity between measured and modeled force production at higher speeds and longer wavelengths.

Speed, *v* (mm/s)	Wavelength, *λ* (mm)	Mean length, *L* (mm)	Std. deviation, *σ*_*L*_ (mm)	$\overline{{\boldsymbol{L}}}$	$\overline{{{\boldsymbol{\sigma }}}_{{\boldsymbol{L}}}}$
80	1	0.088	0.046	0.13	0.055
80	2	0.11	0.060		
80	3	0.19	0.060		
120	1	0.15	0.040	0.19	0.085
120	2	0.14	0.054		
120	3	0.27	0.16		

## Discussion

In this study, we compared sliding friction forces produced by a real finger with those that are predicted via a numerical multi-layer fingertip model that accounts for the geometry, mechanics, and interfacial forces produced by a real finger. The model captured the most salient phenomena that we empirically observed in matched conditions, including quasiperiodic force fluctuations and their dependence on the spatial period of the underlying texture. In measured and simulated forces, the nonlinearity of finger tissue and contact mechanics was reflected in force components that were excited at multiples of the fundamental surface (spatial) frequency (1.0, 0.5, 0.33 mm^−1^).

**Figure 4:**
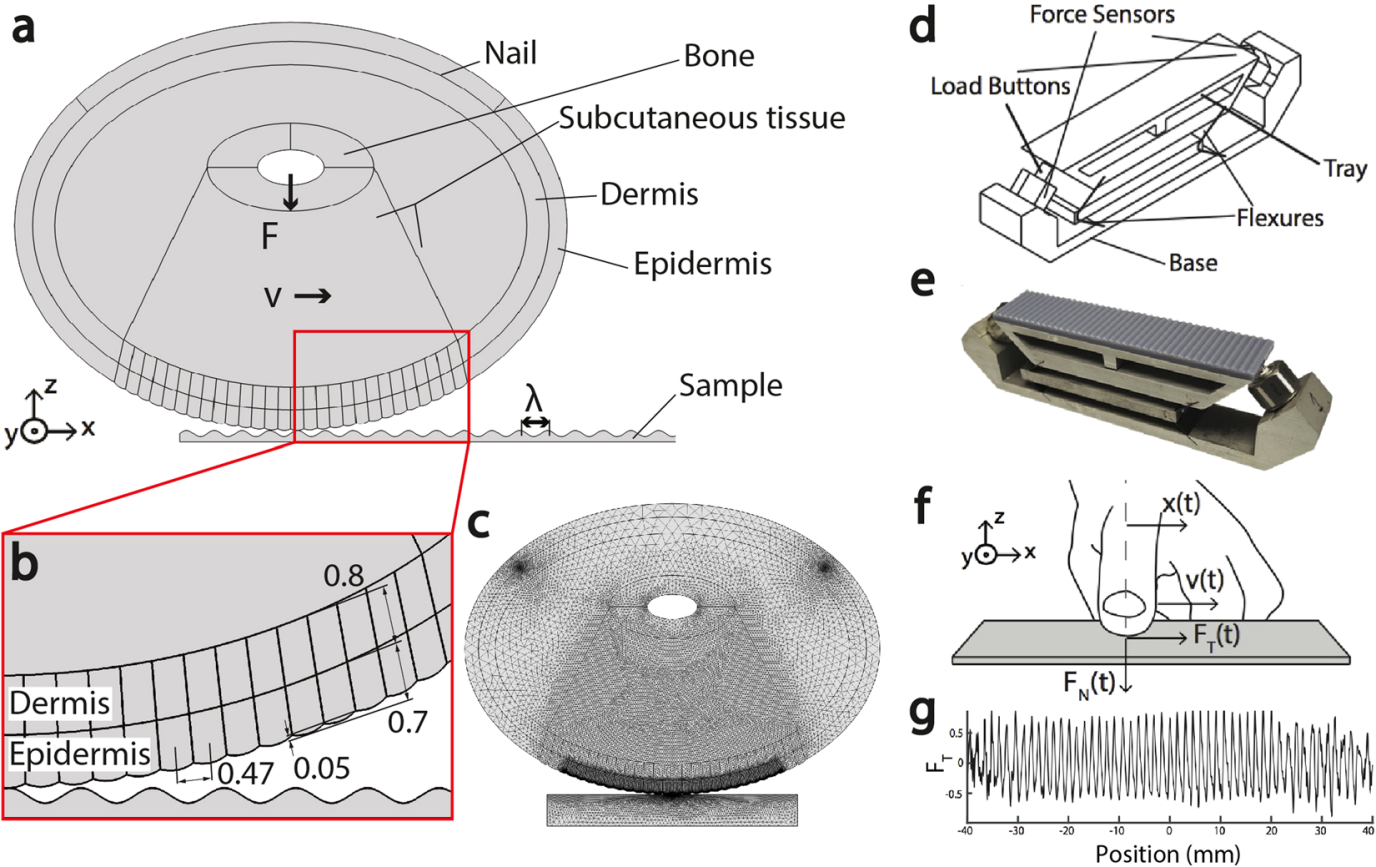
(**a**) Two-dimensional multi-layer FEM model of the fingertip and (**b**) detailed view (mm) sliding over a textured surface with sinusoidal shape. The simulations differ in the surface wavelength (*λ* = 1, 2, 3 mm), fingertip velocity (*v* = 80, 120 mm/s) and the initial fingertip position. The touch force of the fingertip for all six conditions is *F* = 0.3 N. (**c**) Meshing of the fingertip and flat surface into plane stress triangle elements, showing the finer meshing in the contact region of the finger (**d**) Isometric illustration of the apparatus that was used to record real frictional interactions between finger and textured surface (**e**) Description of a finger, sliding over a flat surface fingertip with position x(t), scanning speed v(t), and contact forces *F*_*N*_ and *F*_*T*_ (**f**) Photo of the apparatus with sinusoidal surface sample on top (**g**) Typical example of normalized frictional force signal, produced during sliding of the finger over a sinusoidal grating (*λ* = 1 mm).

Through recurrence analyses, including pairwise comparisons across the ensemble of measured and simulated forces in each condition, we determined that the main features of the measured friction force trajectories were captured by the model, and that the model had greatest predictive power for higher speeds and coarser surfaces. The results were qualitatively highly similar when the analysis was restricted to individual participants (Fig. 4b), and in individual trial analyses for each participant and simulations (see representative examples in Fig. S3). The recurrence analysis revealed greatest similarity between measured and simulated forces (Fig. 3), as quantified by recurrence length statistics, for faster sliding (*v* = 120 mm/s) and larger-scale surface textures (*λ* = 3 mm), see Table 1. This was consistent with our findings from the spectrum analysis. Together, these results suggest that tactile exploration at low speeds on fine surfaces elicits force patterns that are more difficult to predict from such a model, even though they fluctuate more slowly in time. This may partly explain why, as observed nearly a century ago^9^, perceptual discrimination of fine surface texture becomes impaired as the speed of sliding decreases. However, as discussed below, effects of small scale features, including fingerprint ridges further complicate this interpretation.

**Figure 5:**
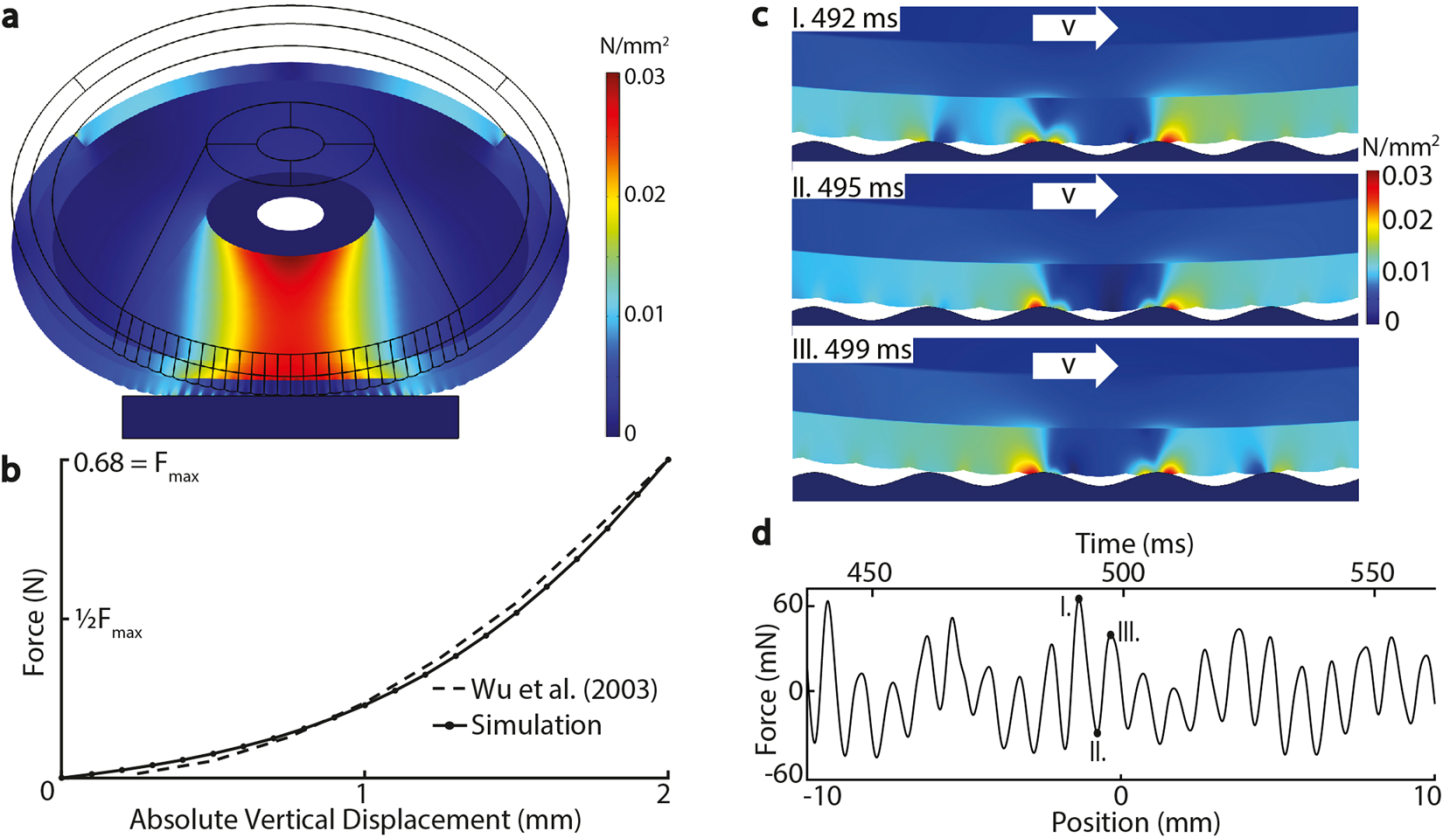
(**a**) The von Mises stress distribution at 2 mm displacement static loading of the fingertip against a rigid plate, illustrating high stress concentrations between bone and surface (**b**) The static nonlinear behavior of a real fingertip is effectively captured by the simulation, as shown by the force-displacement curves (**c**) Von Mises stress distribution for the condition (*λ* = 1 mm, *v* = 80 mm/s) at the time instances I: 492 II: 495 III: 499 ms. High stress values are unevenly present in the contact region of the epidermal layer and attenuate with increasing distance to the finger surface interface. (**d**) Typical simulated force trial (*λ* = 1 mm, *v* = 80 mm/s) as a function of time and space: the oscillating force signal exhibits quasiperiodic behavior with increasing and decreasing amplitude.

At low speed and smaller wavelengths, complex, multiperiod oscillations are observed in measured and simulated forces (Fig. 2), and our linear and nonlinear analyses suggest that this could be attributed to the ability of slow speeds and fine textures to engage small scale features, including finger ridges, in ways that were more complex than are captured in the model. Notably, for slow sliding or finer surface textures, the frequency spectra of the simulated forces included frequency components due to the 0.47 mm fingerprint ridge spacing of the epidermal layer, and apparent mixing frequencies with the surface period. This is due to the effectively parallel geometry of fingerprint ridges in the model relative to the surfaces (see Figs 5 and S4).

While we did not observe such a frequency component in simulated forces for the coarsest surfaces, a closer analysis of the volumetric simulations revealed that out-of-phase motion of epidermal ridges was responsible for this (see Supplementary Information S4). At the slowest speed, this attenuated the frequency component associated with the surface wavelength. A further result was a decrease in bulk stresses which could, in principle, reduce tactile sensation for surfaces that coupled to the dynamics of a real finger in such a manner. This may merit further investigation.

In contrast to these simulations, during tactile exploration of real surfaces, fingerprint ridges are oriented in multiple directions. Viewed along the direction of motion, this results in a range of effective ridge spacings, and thus would be expected to elicit a distribution of force frequencies, rather than a single dominant frequency, as would be consistent with our measurements.

While our experimental observations in this study were dominated by frequencies due to the surface geometry, previous research has reported friction force components associated with spatial frequencies 1/*λ*_*Ridge*_ of the fingerprint ridges^32^, and that such effects can be robust to contact orientation^19^. These effects have been linked to a hypothesized sensory function of finger ridges. As noted above, their absence in our measured data could be due to irregularities in real fingerprint patterns, their nonuniform alignment with the surface texture along the direction of motion, or to the moisture-induced plasticization of fingerprint ridges.

At larger length scales, combined effects of finger elasticity and surface periodicities may also help to explain the higher trial to trial variability of friction forces produced by finer surfaces. For these surfaces, contact conditions change rapidly, yielding many distinct contact regions, and contributing to the complexity of the resulting force patterns^33^. As we have recently shown, such multi-contact surfaces are not only produced during contact with high relief textures, but also generically manifest during tactile exploration of smooth, isolated surface features, such as localized bumps^34^. The gaps in contact that occur during tactile interaction with relief surfaces can enhance bulk stress gradients that are captured by sensory mechanoreceptors of touch, and further research is needed in order to clarify their role in tactile sensation.

While contact-dependence represents a nonlinearity in the system, coupled to the high-dimensional tissue dynamics, other sources of variation in the interfacial or dynamic conditions that were not modeled here could also contribute to force fluctuations during tactile surface exploration. In addition to fingerprint effects discussed above, additional potential sources of variation that arise from the complex tribological behavior of the finger pad^12,13,35^ include the moisture-driven plasticization of the stratum corneum (which we minimized methodologically) and partial stick-slip motions. Further research to investigate these factors is warranted. In addition, the nonlinear dynamics of heterogeneous tissues could yield significant variation in time-resolved friction forces arising from even proximal initial conditions.

## Methods

We investigated the geometric and mechanical origins of force production during frictional sliding of a finger against textured surfaces. We analyzed measurements of time varying contact forces produced as real fingers slid on relief surfaces, numerically simulated the same system, and compared both using signal processing and recurrence plot analysis and quantification. This study was directed at understanding physical phenomena, and did not comprise human research (US Department of Health and Human Services 45 CFR 46.102(f): No identifiable or private data were used or coded).

### Measured Force Production

This study is based on data from a custom force and position measurement apparatus (Fig. 4d,e) from our previous work, in which we collected normal and tangential force data produced while fingers slid over fabricated undulating surfaces^6^, as we summarize here. We measured forces via piezoelectric sensors (Model 9712A5, Kistler Instruments, Switzerland) supporting a textured relief surfaces on an aluminum tray (top dimensions 120 × 25 mm) via a compliant mechanism^36^, and captured the data to maximize the available signal bandwidth after digitization (55.6 *μ*s sample period, 16 bits).

The textured relief surfaces were sinusoidal gratings with surface height function *h*(*x*) = *A* sin (2*πx*/*λ*), which varied in wavelength *λ* and amplitude *A*. Wavelength and amplitude were coupled through a scale parameter, *A*(*λ*) = *A*_0_*λ* with *A*_0_ = 0.1 mm, ensuring that the maximum slope was constant for all surfaces. The range of wavelengths (1 to 3 mm) was similar to those used in previous studies of texture perception from end point forces^37,38^. The surfaces were 3D printed (Objet 30, Stratasys Inc., Boston, USA) with high resolution (100 *μ*m) yielding surfaces that were sinusoidally textured with a smooth finish. Finger motion was tracked using an optical system (model V120: Trio Natural Point, Corvallis, OR), via a reflective marker attached to the fingernail, with sampling rate 120 Hz, spatial resolution 200 *μ*m.

The high resolution force and motion data were produced by the fingers of nine individuals (five male and four female, age range 19 to 28). Digit I (index finger) of the dominant hand slid over the surfaces ten times at two different speeds (80 mm/s and 120 mm/s), as entrained through auditory feedback, from left to right. The finger was not restrained, in order to preserve the mechanical effect of the joints of the hand. The fingers contacted the surface with a touch force of *F* = 0.3 N, as entrained through a visual scale. The two speeds and three wavelengths yielded a total of six conditions. We excluded a small number of trials with periodic or transient stick slip behavior that exhibited large transient force magnitude fluctuations. 88 measured force signals in each condition (*v*, *λ*) were used for the analysis. Prior to data collection in each condition, each sinusoidal grating and finger pad had been cleaned using isopropyl alcohol.

### Numerical Friction Simulation

To clarify the dynamics of frictional sliding during tactile surface exploration, we simulated sliding friction interactions in conditions mirroring those of the real measurements, using FEM software (COMSOL Multiphysics, COMSOL Group, Sweden), inspired by prior numerical studies of finger biotribology^27^. In order to ensure efficient computation, we chose a two-dimensional modeling approach. We constructed an axially symmetric fingertip model, accounting for the mechanics and the main anatomical features of the finger and of the touched surface. The model accommodates the heterogeneity of finger tissues, including the nail, bone, dermis and epidermis (thicknesses 0.8 and 0.7 mm^39,40^), epidermal ridges, and subcutaneous tissues (Fig. 4a,b). We set the center-to-center distance of the ridges to be 0.47 mm, consistent with prior research^41^. We omitted papillae between the dermis and epidermis from the model, as there is little evidence that they play a role in frictional dynamics. We selected material properties, incorporating hyperelasticity, so that the nonlinear constitutive behavior of real fingers under indentation (compression) testing was reconstructed^42^ (Fig. 5 and Supplementary Information S1).

At each time, the numerical model computed bulk and interfacial stresses and strains due to sliding contact between the finger and surface, yielding normal and tangential stress components *σ*_*p*_(*x*) and *σ*_*r*_(*x*) at each point *x* on the contact interface. Their directions correspond to the surface orientation at *x*, given here via the angle *α*(*x*) = tan^−1^ [*dh*(*x*)/*dx*] formed by the tangent of the surface *h*(*x*) and the horizontal. The normal and tangential stress are related by a Coulomb-Amonton term *σ*_*r*_(*x*) = *μσ*_*p*_(*x*), with a coefficient of friction *μ* that is independent of the applied load. This assumption has theoretical and empirical support over a wide range of conditions^43,44^, although at low forces modest variations of *μ* with the normal force applied by a finger are observed^18^. At each time *t*, the friction force on the finger is given by projecting stress components in the direction tangent to the surface and integrating over the contact surface ${\mathscr{C}}$, yielding

1 $$F={\int }_{{\mathscr{C}}}{\sigma }_{p}(x)\{\sin (\alpha (x))+\mu \,\cos \,(\alpha (x))\}dx\,dz$$

where *z* spans a size *D* in the plane of the model.

To match the measurement conditions, the indentation of the finger was controlled to yield a normal force of 0.3 N, and the friction coefficient was set to *μ* = 0.8. Further detail about the model parameters and simulation details are provided in the Supplementary Informations S1–S3.

Prior research^13,35^ including work in our lab^6^, has reported large trial to trial frictional force fluctuations, which were partly attributed to small (≈100 *μ*m) variations in initial contact conditions. In order to capture this source of variability, we repeated simulations from an array of ten initial positions in every condition.

### Linear Analysis of Frictional Forces

We analyzed real and simulated friction force signals *F*_*T*_(*t*) in order to ascertain the factors determining force fluctuations from surface texture. We isolated force signal components of the measured and simulated time series that were most relevant to tactile perception using band pass filtering (zero phase, corner frequencies 15 and 500 Hz). We translated these signals from the time to the spatial domain using the centroid of the finger position *x*(*t*), yielding spatial domain force signals *F*_*T*_(*x*). We analyzed the spatial frequency content in the measured and simulated signals using the Fourier transform, yielding ensembles of frequency domain signals *F*_*T*_(*f*).

### Nonlinear Analysis of Frictional Forces

Linear measures, including Pearson’s cross correlation, can yield low similarity scores between measurement trials in otherwise identical conditions, and are prone to spurious effects. The complexity of the force signals as well as the nonlinearity of the mechanics also raised the question of whether linear methods would capture signal attributes of interest. Thus, we investigated the similarity between measured and simulated frictional signals using recurrence plot analysis, in order to extract multi-mode and nonlinear dependencies, to compare the series across time, and to quantify similarities in the force patterns of simulated and measured trials. The recurrence analysis method embeds the time series *f*_*i*_ = *f*(*t*_*i*_), *i* = 1, 2, …, *N*, into a state space of dimension *D*, in which a state vector trajectory

2 $${{\bf{x}}}_{i}=({f}_{i},\,{f}_{i+\tau },\,\ldots ,\,{f}_{i+(D-1)\tau }),\,i=1,2,\ldots ,K$$

of length *K* = *N* − (*D* − 1)*τ* is obtained as the span of *D* samples each spaced by a delay time *τ* centered at each indexed time *t*_*i*_. The recurrence matrix

3 $${{\bf{R}}}_{ij}(\varepsilon )={\rm{\Theta }}(\varepsilon -\Vert {{\bf{x}}}_{i}-{{\bf{x}}}_{j}\Vert )$$

with *i*, *j* = 1, 2, … *K* describes the similarity of the state at time *t*_*i*_ with the state at a different time *t*_*j*_ and depends on the embedding dimension *D*, delay time *τ*, and a distance threshold value *ε*. Here Θ is the Heaviside step function, ||·|| is the Euclidean distance, and *K* is duration of the state vector. Thus, *R*_*ij*_ is 1 when **x**_*i*_ and **x**_*j*_ are closer than *ε*, and 0 otherwise. To compare state vector trajectories **x**_*i*_ and **y**_*j*_ associated with distinct simulated and measured time series *f*_*i*_ = *f*(*t*_*i*_) and *g*_*j*_ = *g*(*t*_*j*_), we compute a joint recurrence matrix

4 $${{\bf{J}}}_{ij}(\varepsilon )={\rm{\Theta }}({\varepsilon }_{x}-\Vert {{\bf{x}}}_{i}-{{\bf{x}}}_{j}\Vert )\,\cdot \,{\rm{\Theta }}({\varepsilon }_{y}-\Vert {{\bf{y}}}_{i}-{{\bf{y}}}_{j}\Vert )$$

with *i*, *j* = 1, 2, …, *K*, so that all time instances are highlighted, for which two different time series trajectories recur simultaneously in their respective phase space. To analyze ensembles of trials in respective conditions, we computed collective recurrence and joint recurrence matrices

5 $${ {\mathcal R} }_{ij}=\sum _{\alpha =1}^{{N}_{l}}\,{{\bf{R}}}_{ij,\alpha },\,{{\mathscr{J}}}_{ij}=\sum _{\alpha =1}^{{N}_{l}}\sum _{\beta =1}^{{M}_{l}}\,{{\bf{J}}}_{ij,\alpha \beta }$$

with *i*, *j* = 1, 2, …, *K*, accommodating data of *N*_*l*_ = 10 simulated and *N*_*l*_ = 88 measured trials in each condition (*v*, *λ*), yielding a total of parwise 880 combinations. The recurrence matrix **R**_*ij*,*α*_ (Eq. 3) belongs to simulated or measured trial number *α*, and for the joint recurrence matrix **J**_*ij*,*αβ*_ (Eq. 4) *α* and *β* index the measured and simulated trials. We set the distance threshold *ε* adaptively for each **R**_*ij*,*α*_ and **J**_*ij*,*αβ*_ to yield a constant densities of 10 and 35% respectively. The joint recurrence analysis was repeated for the simulated and measured force signals, at each speed (*v* = 80, 120 mm/s) and surface wavelength (*λ* = 1, 2, 3 mm), yielding a total of twelve collective recurrence plots ${ {\mathcal R} }_{ij}(v,\lambda )$, and six collective joint recurrence plots ${{\mathscr{J}}}_{ij}(v,\lambda )$ that comprised 880 measured vs simulated trial combinations. We quantified diagonal structures in each ${{\mathscr{J}}}_{ij}(v,\lambda )$ by computing the distribution *P*(*L*) of line length values, and reported summary statistics in terms of per-condition mean values *L* and standard deviations *σ*_*L*_ for all 880 simulated and measured trial combinations, as well as statistics grouped across speeds. Further details, including the selection of the embedding parameters, are provided in the Supplementary Information S4.

## Electronic supplementary material

Supplementary Information

## Data Availability

The primary data may be retrieved from the following internet address: https://s3.amazonaws.com/rtlab/BK-MJ-YV-Data.zip.
