# Supplementary material for: Complexity, rate, and scale in sliding friction dynamics between a finger and textured surface
Source: Sci Rep. 2018 Sep 12;8:13710. doi: 10.1038/s41598-018-31818-3 (PMC6135846; doi:10.1038/s41598-018-31818-3)
Supplement: Supplementary file 1 — Supplementary Information [file 41598_2018_31818_MOESM1_ESM.pdf]

Complexity, rate, and scale in sliding friction dynamics between a finger and textured surface

Behnam Khojasteh, Marco Janko, Yon Visell

Supplementary Information

Supplementary data 1: Biomechanical parameters of the simulated finger

We used a hyperelastic, five parametric Mooney-Rivlin mechanical model to account for the nonlinear constitutive behavior of the fingerpad. The model encoded the mechanics of the dermis and subcutaneous tissues via a strain energy density function W given by

$$\begin{aligned} W &= C_{10}(\bar{I}_1 - 3) + C_{01}(\bar{I}_2 - 3) + C_{11}(\bar{I}_1 - 3)(\bar{I}_2 - 3) \\ &\quad + C_{20}(\bar{I}_1 - 3)^2 + C_{02}(\bar{I}_2 - 3)^2 + \frac{\kappa}{2}(J_{el} - 1) \end{aligned} \tag{1}$$

Here, $C_{i,j}$ are material parameters, $\bar{I}_1$ and $\bar{I}_2$ are first and second deviatoric strain invariants, J_{el} is the elastic volume ratio and κ is the initial bulk modulus. We determined values (Table S1-1) for the Mooney-Rivlin parameters within ranges from prior literature[1] through numerical optimization, fitting the compression testing force-displacement measurements of Wu et al.[2], in which a flat surface was used to indent the fingerpad, to the model output. The fit minimized a sum-of-squared error residual.

Table S1-1. Material parameter values (kPa) for dermis and subcutaneous tissue

Tissue	C_{10}	C_{01}	C_{11}	C_{20}	C_{02}	$\kappa/2$
Dermis	2.30	5.40	-260	240	750	76.92
Subcut.	0.12	0.91	-66	60	290	9.43

Linear elastic material properties (E, ν) were assigned to the harder tissues, including the nail, bone and the epidermis (Table S1-2), that were subject to smaller displacements using previously reported values[3]. For the sinusoidal grating, we selected material parameters that matched those of the plastic that the real samples were fabricated from ($E = 2.5$ kPa, $\nu = 0.3$).

Table S1-2. Linear material parameters of the fingertip[3]

Layer	E (kPa)	ν	ρ (kg/m^3)
Bone	17000	0.3	1908
Nail	170	0.3	1900
Epidermis	0.08	0.48	1060

The numerical model was discretized with high density to capture stress variations in the finger and surface, using 14,332 plane stress triangle elements (Fig. 4 c). In the contact region, a finer mesh (maximum size 0.05 mm) was used, while a coarser mesh was employed in other regions (element size 0.1 to 0.3 mm). The step size for the sliding simulation was Δt = 1 ms.

Supplementary data 2: Numerical Simulation of Finger Indentation and Sliding Friction Contact

The von Mises stresses under simulated indentation of 2 mm (Fig. 5 a) was highest between the bone and contact surface, with additional stress variations in the region of the nail. Under compression with the optimized biomechanical parameters (Tables S1-1,S1-2) of the simulated finger, we observed close agreement between the nonlinear constitutive response of this model and previously published experimental measurements (Fig. 5b in the paper). During the early loading stage, the soft tissues of the fingertip are quite soft, having approximately 20% of F_{max} at 50% of the maximum displacement.

During sliding friction contact, stress is unevenly distributed within the fingerpad, and the spatial stress distribution fluctuates rapidly in time (Fig. 5 c). The most prominent variations occurred in the epidermal layer, due to the direct interaction with the contacting surface. At all times, stress concentrations are observed to decay with increasing distance from the vicinity of contact.

Supplementary data 3: Sensitivity study of the friction coefficient

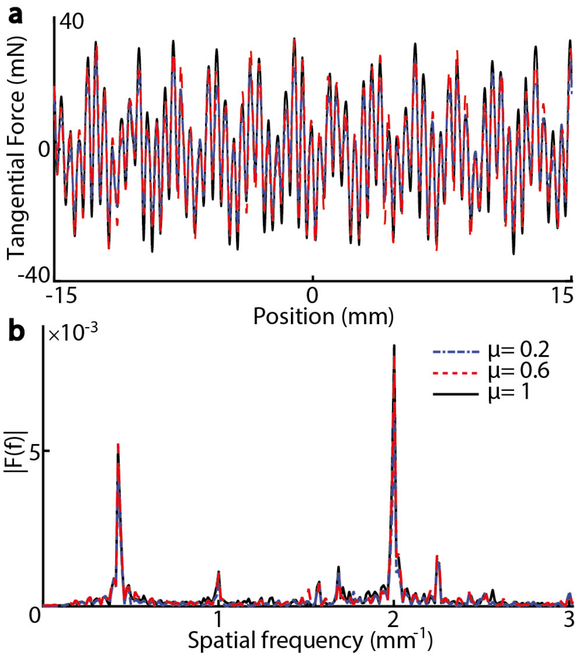

Figure S2. (**a**) Tangential force F_t for three different values of the friction coefficient, that is $\mu = 0.2, 0.6, 1$ in the spatial domain for the condition ($\lambda = 1$ mm, $v = 80$ mm/s). These force signals essentially differ in their force amplitude level due to the Coulomb friction law. (**b**) Magnitude spatial frequency response for the three frictional force signals. The locations and magnitudes of the maxima in the spectrum occur at virtually identical frequencies, with nearly identical amplitudes, indicating little dependence of the spectrum on the value of μ.

For biological systems, the friction coefficient μ can be difficult to identify, and, indeed, can hardly be considered to be constant[4]. In order to determine the effect this parameter would have on spectral content in simulated tangential forces F_t, we selected a representative condition ($\lambda = 1$ mm, $v = 80$ mm/s), simulated sliding contact for three different values of the friction coefficient ($\mu = 0.2, 0.6, 1.0$) in a local Coulomb friction model. We compared spatial force patterns and the magnitude spatial frequency response of the data for each value of μ (Fig.S2). Although the magnitude of friction mostly scales in proportion with μ, we found that the magnitude spectra for different values of the friction coefficient were nearly identical, with principal and secondary maxima occurring at virtually identical frequencies with identical amplitudes. This suggested that the spatial frequency content of sliding friction forces was highly robust to variations in friction coefficient, and was instead determined by other factors, such as surface geometry and bulk mechanics. We selected a value $\mu = 0.8$ for the remainder of the experiments.

Supplementary data 4: Embedding, Quantification and Single Trial Results of Recurrence Analysis

We used a toolbox[5] for the recurrence analysis. In the course of the recurrence plot analysis, proper values of the embedding parameters are required. In order to select values for the embedding parameters, τ and D, that capture the dynamics without

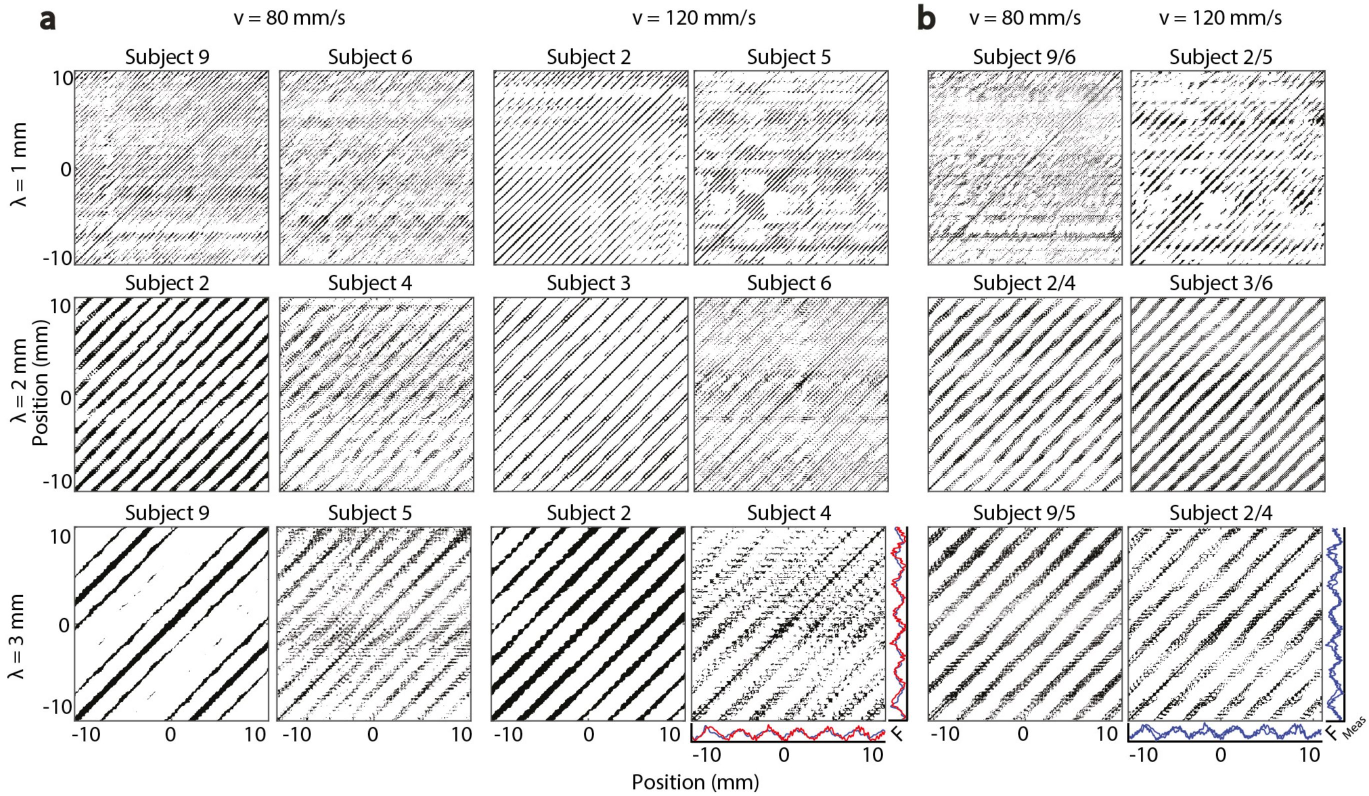

Figure S3. (**a**) Two Single-Trial JRPs in each condition (v, λ) of measured vs. simulated signal pairs from different subjects with low and high agreement in the force dynamics. (**b**) For every condition both measured trials of the corresponding subjects are compared to each other by means of a Single-Trial JRP. While for certain subjects a high similarity between measured and simulated signals prevails, JRPs of other subjects indicate that one fingertip model is not sufficient to cover the frictional force dynamics from the variety of human fingers. Moreover, even though trials from different subjects are governed by the surface wavelength in the respective condition, remarkable differences exist in the force dynamics of the measurements, indicated by the singularities in the measured vs. measured JRPs.

introducing artifacts, we used time-delayed mutual information and the false nearest neighbors (FNN) method[6]. The mutual information as a well-established measure detects nonlinear dependencies without autocorrelated effects within time series. It quantifies the amount of information about one variable that is obtained through another. Time-delayed mutual information $I(\tau)$ of a system is computed via

$$I(\tau) = -2ln\sum_{k} p_k + ln\sum_{k,l} p_{k,l}(\tau) \ , \tag{2}$$

where p_k is the probability to find a time series value of $f(t)$ in the kth time interval and $p_{k,l}(\tau)$ is the joint probability that $f(t)$ falls into the kth as well as $f(t+\tau)$ into the lth time step. The first local minimum in $I(\tau)$ provides an estimate for the time delay value, τ.

The core idea of the FNN method, for which Rhodes et al.[7] describe the algorithm, is de is to examine how the number of neighbors of a point along a signal trajectory change as a function of the embedding dimension D. With increasing embedding dimension false neighbors of a trajectory, which are only close to the point because of projection rather than the system dynamics, diminish and by establishing an appropriate embedding, only real neighbors remain. We chose an embedding dimension D, for which no false nearest neighbors existed. For the analysis of the collective recurrence matrices, we set fixed values of τ and D equal to the median estimates for the ensemble of analyzed trials for each $\mathbf{R}_{ij,\alpha}$, and adapted the embedding parameters from simulated or measured data set with higher embedding dimension D for each $\mathbf{J}_{ij,\alpha\beta}$.

In the course of the recurrence analysis, we identified diagonal structures in the joint recurrence matrices, to quantify similar dynamical trends in the simulated and measured time series. In general, diagonal structures in recurrence plots imply that two segments of a trajectory are relatively close to each other, and respectively exhibit a measure related to the divergence of trajectory segments in a time series. In a joint recurrence plot, diagonal structures represent simultaneous occurrences of two

trajectories and therefore quantify a similar time evolution in terms of dynamics in both time series. The average length of diagonal lines is given by

$$h_{\alpha\beta} = \frac{\sum_{l=l_{min}}^{N} lP(l)}{\sum_{l=l_{min}}^{N} P(l)} \;, \tag{3}$$

where $P(l)$ denotes the probability to find a diagonal line of length l in the joint recurrence plot, corresponding to time series trajectories α and β. Since the force signals exhibit strong fluctuations, we considered line segments, consisting of two points ($l_{min} = 2$) or more in order to accommodate for joint recurrences with even small duration for the quantification analysis.

JRP Examples from individual trials that were consistent with the average behavior and those that were very different from the average behavior, illustrate the contrast of friction-induced forces for different human finger. The JRPs from measured vs. measured signal pairs also show that for the same condition different subjects can produce significantly differing frictional forces, especially at lower surface wavelengths.

Supplementary data 5: Inter-Ridge Phase Cancellations

The surface period governs the fundamental frequency of the measured force signals, whereas for the numerical simulation the respective frequency component is attenuated in all conditions (v,λ), because the epidermal ridges are driven out of phase, leading to a reduction in resultant force at the frequency component of the textured surface. The frequency magnitude response (A,C) and phase spectrum (d) of single-ridge force contributions for the condition ($\lambda = 3$ mm,$v = 80$ mm/s) exemplify this out-of-phase behavior (Fig. S4). The phase spectrum reveals that the fundamental frequency of the sample, given by 0.33 mm^{-1} corresponds to the out-of-phase motion between different ridges and the frequency peak at 0.41 mm^{-1} to the in-phase motion.

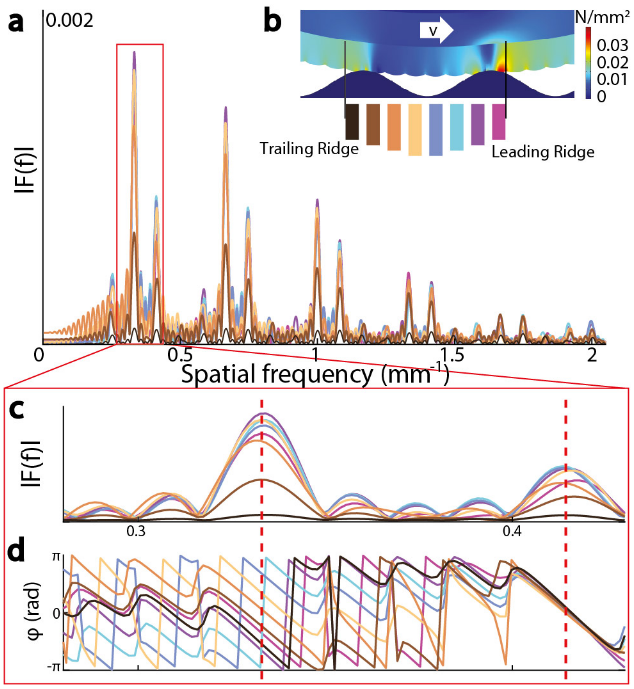

Figure S4. (**a**) Magnitude spectrum of the simulated forces over individual ridges for the condition ($\lambda = 3$ mm,$v = 80$ mm/s) with corresponding (**b**) von Mises stress distribution for a time instance and legend of the epidermal ridge force contributions. The (**c**) zoomed-in view of the FFT and the corresponding (**d**) phase spectrum illustrate that the ridges are driven out of motion for the surface frequency component 0.33 mm^{-1} and are in phase for the spatial frequency 0.41 mm^{-1}. These kind of phase cancellations occurs in all simulated conditions (v,λ), leading to an attenuation of the surface frequency component for the accumulated contact force.

Therefore the spatial frequency of the surface period and its harmonics at integer frequency multiples are salient in the magnitude spectrum for forces over individual ridges, but due to phase cancellations are attenuated in the spectrum for the sum of ridge contact forces. The magnitude spectrum of forces over individual ridges also exhibits phase shifts in the spatial

frequency distribution, which may be due to non-equidistant ridge spacings as should be expected by the asymmetrical strain distribution during tactile exploration.

References

1. Wu, J. Z., Welcome, D. E. & Dong, R. G. Three-dimensional finite element simulations of the mechanical response of the fingertip to static and dynamic compressions. *Comput. Methods Biomech. Biomed. Eng.* **9**, 55–63 (2006).
2. Wu, J. Z., Dong, R. G., Smutz, W. & Rakheja, S. Dynamic interaction between a fingerpad and a flat surface: experiments and analysis. *Med. engineering & physics* **25**, 397–406 (2003).
3. Shao, F., Childs, T. H. & Henson, B. Developing an artificial fingertip with human friction properties. *Tribol. Int.* **42**, 1575–1581 (2009).
4. Derler, S. & Gerhardt, L.-C. Tribology of skin: review and analysis of experimental results for the friction coefficient of human skin. *Tribol. Lett.* **45**, 1–27 (2012).
5. Marwan, N. Cross recurrence plot toolbox for matlab (r2016b) (2004). URL http://tocsy.pik-potsdam.de/CRPtoolbox/.
6. Kantz, H. & Schreiber, T. Nonlinear time series analysis, vol. 7 of cambridge nonlinear science series (1997).
7. Rhodes, C. & Morari, M. The false nearest neighbors algorithm: An overview. *Comput. & Chem. Eng.* **21**, S1149–S1154 (1997).
